# PML nuclear bodies contribute to the basal expression of the mTOR inhibitor DDIT4

**DOI:** 10.1038/srep45038

**Published:** 2017-03-23

**Authors:** Jayme Salsman, Alex Stathakis, Ellen Parker, Dudley Chung, Livia E. Anthes, Kara L. Koskowich, Sara Lahsaee, Daniel Gaston, Kimberly R. Kukurba, Kevin S. Smith, Ian C. Chute, Daniel Léger, Laura D. Frost, Stephen B. Montgomery, Stephen M. Lewis, Christopher Eskiw, Graham Dellaire

**Affiliations:** 1 Department of Pathology, Dalhousie University, Halifax, Nova Scotia, B3H 4R2, Canada; 2 Department of Pathology and Laboratory Medicine, Nova Scotia Health Authority, Halifax, Nova Scotia, B3H 1V8, Canada; 3 Departments of Genetics and Pathology, Stanford University, Stanford, California, 94305, USA; 4 Atlantic Cancer Research Institute, Moncton, New Brunswick, E1C 8X3, Canada; 5 Department of Microbiology & Immunology, Dalhousie University, Halifax Nova Scotia, B3H 4R2, Canada; 6 Department of Chemistry & Biochemistry, Université de Moncton, Moncton, New Brunswick, E1A 3E9, Canada; 7 Department of Biology, University of New Brunswick, Saint John, New Brunswick, E2L 4L5, Canada; 8 Department of Department of Food and Bioproduct Sciences, University of Saskatchewan, Saskatoon, Saskatchewan, S7N5A8, Canada; 9 Department of Biochemistry & Molecular Biology, Dalhousie University, Halifax, Nova Scotia, B3H 4R2, Canada; 10 Beatrice Hunter Cancer Research Institute, Halifax, Nova Scotia, B3H 4R2, Canada

## Abstract

The promyelocytic leukemia (PML) protein is an essential component of PML nuclear bodies (PML NBs) frequently lost in cancer. PML NBs coordinate chromosomal regions via modification of nuclear proteins that in turn may regulate genes in the vicinity of these bodies. However, few PML NB-associated genes have been identified. PML and PML NBs can also regulate mTOR and cell fate decisions in response to cellular stresses. We now demonstrate that PML depletion in U2OS cells or TERT-immortalized normal human diploid fibroblasts results in decreased expression of the mTOR inhibitor DDIT4 (REDD1). DNA and RNA immuno-FISH reveal that PML NBs are closely associated with actively transcribed *DDIT4* loci, implicating these bodies in regulation of basal DDIT4 expression. Although PML silencing did reduce the sensitivity of U2OS cells to metabolic stress induced by metformin, PML loss did not inhibit the upregulation of DDIT4 in response to metformin, hypoxia-like (CoCl_2_) or genotoxic stress. Analysis of publicly available cancer data also revealed a significant correlation between *PML* and *DDIT4* expression in several cancer types (e.g. lung, breast, prostate). Thus, these findings uncover a novel mechanism by which PML loss may contribute to mTOR activation and cancer progression via dysregulation of basal DDIT4 gene expression.

The promyelocytic leukemia (PML) protein is a tumor suppressor and reduced PML expression is associated with several cancers (e.g. breast, prostate and lung)^1^. Mice that lack PML are more prone to cancer in response to mutagens^2^ or when bred with mice lacking the PTEN tumour suppressor^3^. PML is the structural component of subnuclear domains known as PML nuclear bodies (PML NBs), which are dynamic heterogeneous protein complexes that regulate cell stress responses and cell fate decisions (i.e. apoptosis, cell division, senescence) (reviewed in refs 4, 5 and 6). Many of the more than 150 nuclear proteins known to associate with PML are implicated in gene regulation including transcription factors (TFs) (e.g. p53, STATs, SP1), TF regulators (e.g. pRb, CK1, HIPK2, SENPs) and chromatin modifiers (e.g. HDACs, Daxx)^4^,^7^,^8^. Although devoid of nucleic acids, PML NBs make extensive contacts with chromatin^9^, and are associated with specific gene loci such as the *TAP1*^10^ and the *TP53* gene^11^. Although few specific NB-associated gene interactions are known, PML NBs can associate with transcriptionally active chromosomal regions^12^,^13^, implicating active transcription and/or TFs (or other DNA binding proteins) in driving the association of specific loci with these NBs. Although the specific role of PML NBs in gene regulation is unclear, a model has emerged where PML NBs serve as organizing centers or “hubs” for nuclear protein sequestration and post-translational modification (e.g. phosphorylation, SUMOylation, ubiquitination, acetylation)^14^. For example, PML NBs play a role in p53-mediated transcription by promoting the activation and stabilization of this TF through post-translational modifications including acetylation^15^,^16^, phosphorylation^17^,^18^ and SUMOylation^19^.

PML also regulates cell growth, stress responses and tumor suppression through a complex relationship with the mechanistic target of rapamycin (mTOR). Under stress conditions, mTOR activity is inhibited; which in turn, inhibits cell proliferation and activation of energy generating pathways, such as autophagy and glycolysis. Under hypoxia PML can directly inhibit mTOR by sequestration at PML NBs, which in turn impairs the induction of HIF1α^20^. In response to growth factors or oncogenic RAS, PML can inhibit mTOR by inactivating phospho-AKT by co-recruitment of AKT and its phosphatase PP2A to PML NBs^3^. PML loss compounded with loss of tuberous sclerosis complex (TSC) 2, an mTOR inhibitor, can also increase the activation of mTOR complex 1 (mTORC1)^21^. Thus through various pathways, direct (mTOR) and indirect (PP2A/AKT), PML loss activates mTOR. In this study we have made the novel discovery that expression of another regulator of mTOR, the DNA damage inducible transcript 4 (DDIT4), is dysregulated by loss of PML.

DDIT4 positively regulates the mTOR inhibiting tuberous sclerosis complex (TCS1/2)^22^, and is upregulated in response to various cellular stresses, including DNA damage^23^, hypoxia^24^,^25^ and energy stress^26^. A number of TFs are known to regulate the stress-induced expression of DDIT4 including p53, SP1 and HIF1α^23^,^27^. Given that DDIT4 upregulation occurs in response to hypoxic and genotoxic stress, and that PML is known to regulate these same processes, in part through changes in gene expression, we investigated whether DDIT4 was also regulated by PML. We demonstrate here for the first time that PML loss leads to reduced basal DDIT4 expression, which can, in turn alter DDIT4-dependent effects on mTOR activation following various cellular stresses. We also demonstrate that the chromosomal locus containing the *DDIT4* gene is directly associated with PML NBs, and that DDIT4 and PML expression levels are significantly correlated in several solid tumours including breast, lung and ovarian cancers.

## Results

### PML loss results in lower DDIT4 expression

**Figure 1:**
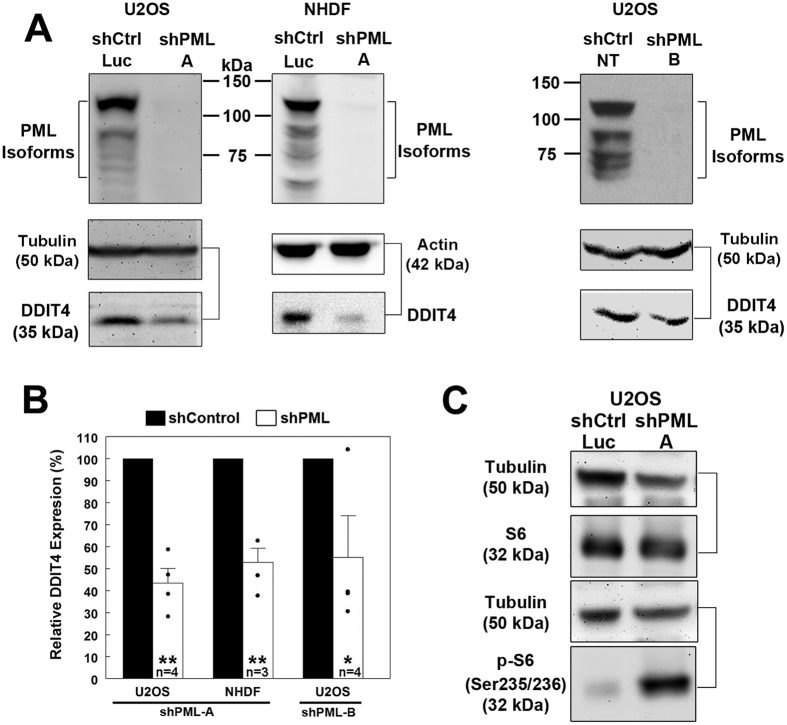
PML silencing is associated with decreased DDIT4 expression. (**A**) Western blot analysis of PML and DDIT4 protein expression in U2OS and NHDF cells expressing control shRNA targeting luciferase (shCtrl-Luc), non-targeting shRNA (shCtrl-NT) or shRNA targeting PML (shPML **A**, shPML B). (**B**) Relative protein expression in shPML cells from Western blots described in **A**. Values represent the mean+/−SEM. *p-*values < 0.01 (**) or < 0.05 (*). (**C**) Western blot analysis for S6 and phospho(Ser235/236) S6 levels in shCtrl-Luc (shControl) and shPML A (shPML) U2OS cells. Full-length blots for this figure are presented in Supplementary Figure 3.

In order to investigate if PML might also regulate DDIT4, we first generated two PML knock-down cell lines. PML was stably silenced in U2OS osteosarcoma cells and TERT-immortalized normal human diploid fibroblasts (NHDF) using a previously well characterized short-hairpin RNA^28^ (shPML-A; Fig. 1A). In both cells lines, silencing of PML was associated with decreased levels of DDIT4 protein (Fig. 1A), with U2OS cells showing a greater decrease than NHDF cells (57% vs 47%, Fig. 1B). To control for off-target effects with the shPML hairpin, we also silenced PML in U2OS cells using a second commercially available short-hairpin RNA (shPML-B) and observed a similar decrease (45%) in DDIT4 expression by Western blotting (Fig. 1A and B). Since the U2OS shPML-A showed a greater decrease in DDIT4 expression than the shPML-B cells, we used the shPML-A U2OS cell line for all subsequent experiments unless otherwise indicated.

Activation of mTOR leads to phosphorylation of S6-Kinase, which subsequently phosphorylates and activates the ribosomal protein S6 at serine residues 235/236^29^. Given that DDIT4 is an inhibitor of mTOR activity, we next assessed whether PML knock-down affected the phosphorylation status of S6 in our cell system, and indeed we observed an increase in S6 phosphorylation in shPML cells that correlated with reduced DDIT4 expression (Fig. 1C). These results are consistent with previous work linking PML loss to increased phospho-S6^20^. However, our new data extends these findings to include reduced DDIT4 expression as a contributing mechanism to mTOR activation following the loss of PML.

### The DDIT4 protein is more stable in PML-depleted cells

**Figure 2:**
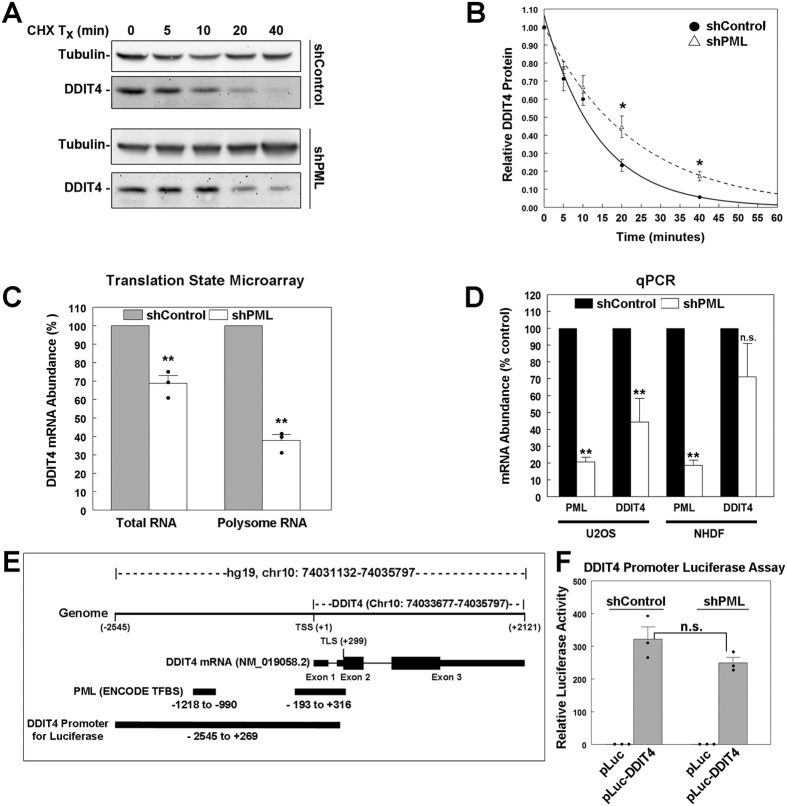
PML loss inhibits DDIT4 gene expression. (**A**) Western blot analysis of DDIT4 expression in shControl and shPML U2OS cells treated with cycloheximide (CHX) for the indicated times. Representative blots from 3 independent experiments were chosen to best illustrate the differences in protein stability as quantified in (**B**). Unprocessed full-length blots for this figure are presented in Supplementary Figure 4. (**B**) Quantification of DDIT4 protein levels over time in cells treated as in **A**. For each cell line, DDIT4 protein densitometry values were normalized to tubulin and are reported relative to the t_0_ time point (n = 3). (**C**) Quantification of DDIT4 total RNA and polysome-associated RNA from shControl and shPML U2OS cells (n = 3). (**D**) QRT-PCR of DDIT4 and PML mRNA abundance in U2OS (n = 5) and NHDF (n = 7) for shPML and shControl cells. (**E**) DDIT4 genomic region (hg19, chr10: 74031132–74035797). The DDIT4 transcript (NM_019058.2) contains three exons and an ORF spanning exons 2 and 3 (thick lines). PML binding sites are indicated (source: ENCODE, transcription factor binding site (TFBS) data), as well as the ~2.7 kb promoter region used in F is indicated. TSS = transcription start site, TLS = translation start site. (**F**) Quantification of luciferase expression in U2OS shControl and shPML cells (n = 3) driven by the DDIT4 promoter indicated in **E**. Values are the mean+/−SEM. *p-*values < 0.01 (**) or < 0.05 (*) and n.s. = not significant. Full-length blots for this figure are presented in Supplementary Figure 4.

We next investigated possible explanations for altered DDIT4 protein expression in the shPML cells, which could include reduced transcription, translation, and/or changes in protein stability. DDIT4 is subject to post-translational regulation through ubiquitination and degradation through the 26S proteasomal pathway and has a reported half-life of ~5–10 min under normal growth conditions^30^,^31^,^32^. Using cycloheximide to block *de novo* protein synthesis, we observed the degradation of DDIT4 over 40 min in U2OS shControl and shPML cells and found that DDIT4 appeared to be more stable in the shPML cells (Fig. 2A). In addition, we performed three independent experiments and calculated a decay curve for DDIT4 protein loss in the shControl and shPML cells (Fig. 2B). Despite DDIT4 protein levels being lower in PML-depleted cells (Fig. 1), the DDIT4 protein was significantly more stable at 20 and 40 min in the shPML cells with a half-life of ~16 min compared to ~10 min in shControl cells. Thus, the reduced DDIT4 levels seen in the shPML cells are not due to increased degradation of DDIT4.

### Loss of PML protein is associated with decreased DDIT4 transcription rather than altered translation

Since reduced DDIT4 protein expression in PML depleted cells cannot be explained by altered protein stability, we sought to determine if PML loss affected DDIT4 transcription and/or translation. To address these questions we used whole genome microarrays to assess the gene expression changes in U2OS shControl and shPML cells. In addition, since PML is known to associate with mTOR^20^ and various components of the translation initiation machinery including eIF3E^33^, eIF3K^34^ and eIF4E^35^, we also assessed differences in translation by identifying the genes with altered association with the polyribosome fraction (translation state array)^36^. Using these approaches, we identified DDIT4 as significantly depleted in both the total RNA and polysome fractions of the shPML cells (*p* < 0.01; Fig. 2C). Since DDIT4 transcripts were reduced in the total RNA fraction in shPML U2OS cells compared to shControl cells, altered transcription rather than translation is likely responsible for the reduced DDIT4 transcripts in the polysome fraction. Consistent with these data, we confirmed that total DDIT4 gene expression was significantly impaired in the shPML U2OS cells by quantitative reverse transcription- PCR (qRT-PCR) (*p* < 0.01; Fig. 2D). We also assessed DDIT4 mRNA expression in NHDF cells, which have reduced DDIT4 protein expression with PML silencing (Fig. 1). Although not statistically significant, we did observe a trend towards reduced DDIT4 mRNA in the shPML NHDF cells (*p* = 0.19) (Fig. 2D). These results indicate that PML loss is associated with decreased *DDIT4* gene expression rather than altered translation. Given the robust and reproducible changes in *DDIT4* gene expression with PML loss in U2OS cells, all further investigation of the transcriptional regulation of *DDIT4* by PML employed this cell line.

In order to address how transcription of the *DDIT4* gene might be affected by PML loss, we cloned a 1683 bp fragment (−1561 to +122) of the *DDIT4* promoter from U2OS cells into a luciferase expression system (Fig. 2E). This DNA fragment encodes several reported regulatory elements for DDIT4 transcription in response to various stimuli, including IR and hypoxia. For example, a p53 response element is located about 300 bp upstream of the TSS and in SAOS2 and U2OS cells this is important for p53-dependent activation of *DDIT4* in response to ionizing radiation^23^. As well, *DDIT4* induction during hypoxia depends on an SP1 site located at −474 to −446 and a hypoxia response element (HIF1α binding) located −427 to −407^27^,^37^. We also analyzed the *DDIT4* promoter region (−2500 to +500) for potential association with PML using the ENCODE transcription factor binding data available through UCSC genome browser (https://genome.ucsc.edu/) and found two regions within our cloned *DDIT4* promoter sequence that were associated with PML by ChIP-Seq (Fig. 2E). The shorter region (peak 1, −1218 to −990) was identified in K562 cells and had a ChIP-seq peak signal score of 778/1000. The longer region (peak 2, −193 to +316) was identified in both K562 and GM12878 cells ChIP-Seq signal scores of 925/1000 and 1000/1000 respectively and our cloned promoter fragment contains the sequences from −193 to +122 of peak 2, which overlaps the transcription start site (TSS) of *DDIT4* (Fig. 2E). The expression vector containing the 1683 bp *DDIT4* promoter region robustly induce luciferase expression 300-fold compared to promoterless pLuc control vector in the shControl cells (Fig. 2F), indicating that we had cloned an active promoter. In the shPML U2OS cells, the same reporter induced luciferase activity 250-fold compared to the promoterless control. While the luciferase activity in the shPML U2OS cells tended to be lower than in the shControl cells, this trend was not statistically significant (*p-*value = 0.15). Thus, loss of PML only modestly impacted *DDIT4* promoter activity when assessed in our extra-chromosomal luciferase reporter assay. Since PML is not known to act directly as a TF, these results indicate that the significant changes in endogenous *DDIT4* transcription triggered by PML loss may be occurring at the level of chromatin and/or subnuclear chromosome organization, where PML NBs may play a role in organizing chromosomal domains^14^.

### PML nuclear bodies are associated with DDIT4 gene loci

PML NBs are known to associate with sites of active transcription and at least one gene, *TAP1,* is transcriptionally regulated through association with PML NBs^11^,^12^. Other genes, such as *BCL2*, show no association with PML NBs^11^,^12^,^13^. In the Ching *et al*., study^11^, the relative localization of the *TAP1* and *BCL2* genes relative to PML NBs was determined using immuno-fluorescent *in situ* hybridization (FISH) in cell nuclei fixed to preserve their 3D organization. Therefore, we wanted to employ 3D immuno-FISH to study the relative localization of the *DDIT4* gene loci to PML NBs as compared to *TAP1* (positive control) and *BCL2* (negative control). However, before beginning our 3D immuno-FISH studies, we first examined the relative expression of *DDIT4, PML, BCL2* and *TAP1* between shControl and shPML U2OS cells using RNA-Sequencing (Fig. S1A). The reduced *PML* and *DDIT4* expression in shPML cells based on fold-change relative to shControl cells was very similar to the qRT-PCR data we obtained in Fig. 2D for U2OS cells. We also confirmed *BCL2* and *TAP1* gene expression changes between shControl and shPML U2OS cells by qRT-PCR (Fig. S1B). *TAP1* and *DDIT4* gene expression were, respectively, upregulated or downregulated by PML loss, consistent with a role for PML NBs in regulating these genes. In contrast, loss of PML did not affect *BCL2* expression in U2OS cells.

**Figure 3:**
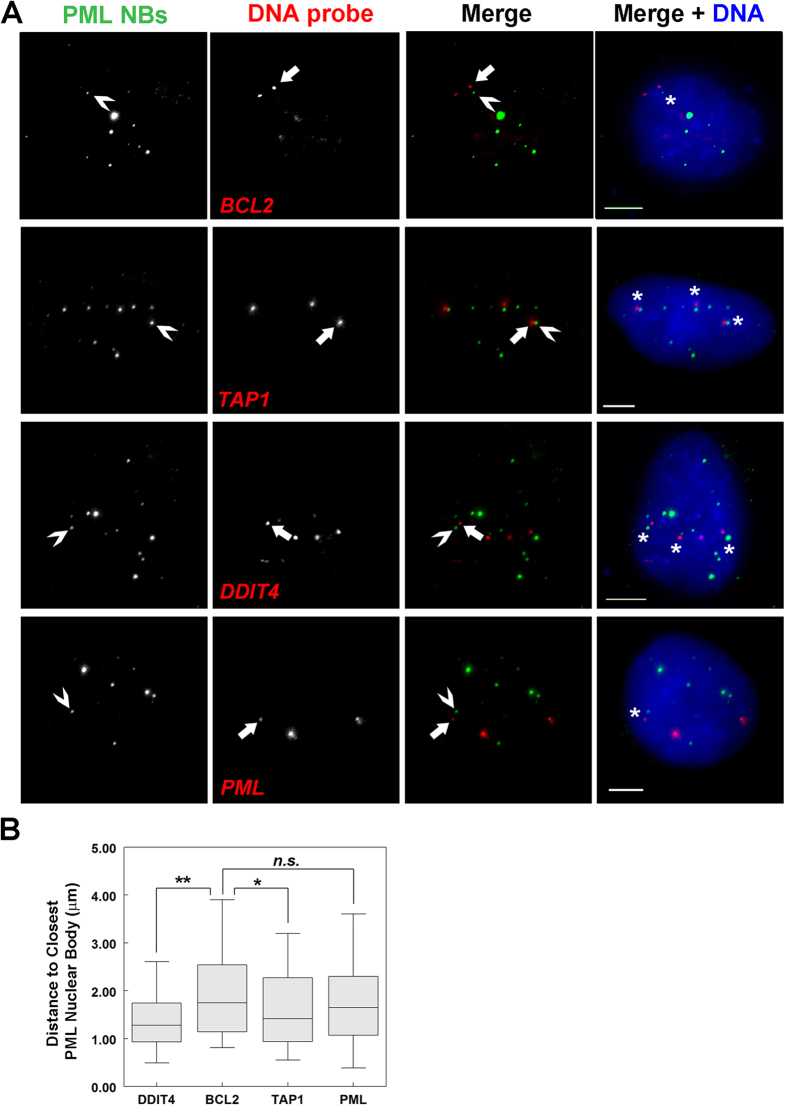
DDIT4 gene loci are proximal to PML NBs. (**A**) U2OS cells were fixed and prepared for 3D immuno-FISH. DNA probes (red) detect the genomic regions containing DDIT4, BCL2, TAP1 and PML; antibodies detect PML within the PML NBs (green), and DNA was visualized with DAPI (blue). In each panel, Arrows indicate the gene loci (red) in closest proximity to a PML NB (green) indicated by a chevron (**>**). *Gene loci within 2 μm of a PML NB. Scale bar = 5 μm. (**B**) Box and whisker plots showing the median and distribution of shortest Euclidean distances between indicated gene loci and the closest PML NB per cell (n = 50 nuclei). *p-*values < 0.01 (**) or <0.05 (*) and n.s. = not significant.

**Figure 4:**
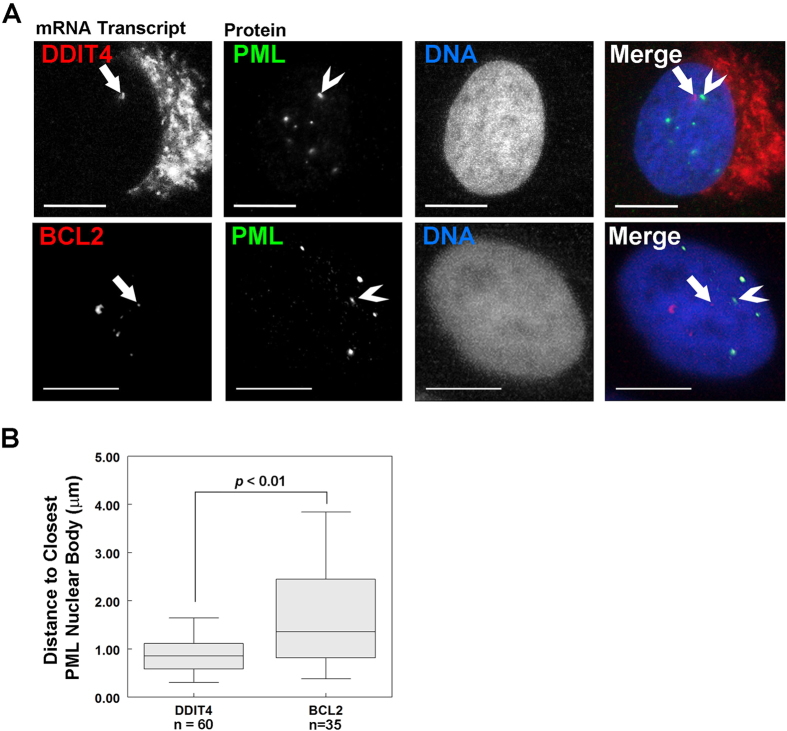
DDIT4 gene expression occurs near PML NBs. (**A**) U2OS cells were fixed and prepared for RNA FISH. DDIT4 and BCL2 mRNA (red) and PML NBs (green) are shown. DNA was visualized with Hoechst 33342. Arrows indicate transcriptional foci (red) and chevrons (**>**) indicate the nearest PML NB (green) to the foci. Scale bar = 5 μm. (**B**) Box and whisker plots showing the median and distribution of shortest distances between a PML NB and the indicated transcriptional foci (n = 30 nuclei).

Having characterized the effects of PML loss on the expression of *TAP1* and *BCL2* in U2OS cells, we next proceeded to 3D immuno-FISH analysis of wild-type U2OS cells using fluorescently labelled BAC probes to detect chromosomal regions containing the *DDIT4, PML, BCL2* and the *TAP1* genes relative to PML NBs (Fig. 3A). PML NBs appeared to be in closer proximity to the *DDIT4* loci than any of the other gene loci; including *TAP1*, which was used as a positive control. In addition, we did not consistently observe PML NBs in close proximity to PML loci, indicating that, in U2OS cells, PML NBs may not regulate PML expression. Using imaging software to visualize in 3D the spatial relationship between PML NBs and each gene loci, we calculated the shortest Euclidian distances between each gene probe and the nearest PML body to provide an average gene probe-PML NB distance per nuclei. This analysis revealed that *TAP1* was significantly closer (average 1.6 μm) to PML NBs than the negative control *BCL2* (average 2.0 μm, *p*-value < 0.05, Fig. 3B). The *DDIT4* gene locus was also significantly closer to PML NBs than BCL2 (1.4 μm, *p*-value < 0.0003). Consistent with our visual assessment, the PML locus was not significantly closer to PML NBs than BCL2 (1.8 μm, *p*-value = 0.22) in U2OS cells. These distances are consistent with previous studies using similar 2D and 3D immuno-FISH methods, where a positive association between gene loci and PML NBs ranged from ~1–1.6 μm and unassociated genes were found at distances of 2 μm or greater from a PML NB^10^,^11^,^12^. Given the previous observation that PML NBs are associated with actively transcribing genes^12^, we employed immuno-RNA FISH to determine if PML NBs are associated with *DDIT4* RNA transcriptional foci (Fig. 4A). In these studies we used *BCL2* RNA-FISH as a negative control since PML loss did not affect *BCL2* expression (Fig. S1). Similar to the immuno-DNA-FISH studies, we calculated the distance between each *DDIT4* or *BCL2* RNA focus to the closest PML NB (Fig. 4A). We found that *BCL2* transcriptional foci were on average 1.7 μm from a PML NB, mirroring the DNA FISH results; whereas, *DDIT4* transcriptional foci were on average 1.1 μm from the nearest PML NB, which is significantly closer than *BCL2 (p-*value < 0.0001; Fig. 4B). We also noted that the RNA-FISH focus to PML NB distances for *DDIT4* RNA foci were much smaller than for *BCL2* foci (Fig. 4B), and were closer than observed for *DDIT4* DNA FISH (Fig. 3), which suggests that actively transcribed *DDIT4* loci are more closely associated with PML NBs. Together, these data support a model in which PML NBs are associated with actively transcribing *DDIT4* gene loci in U2OS cells and this association supports a role for PML NBs in the regulation of *DDIT4* gene expression.

### Effects of PML silencing on DDIT4 induction by CoCl_2_

**Figure 5:**
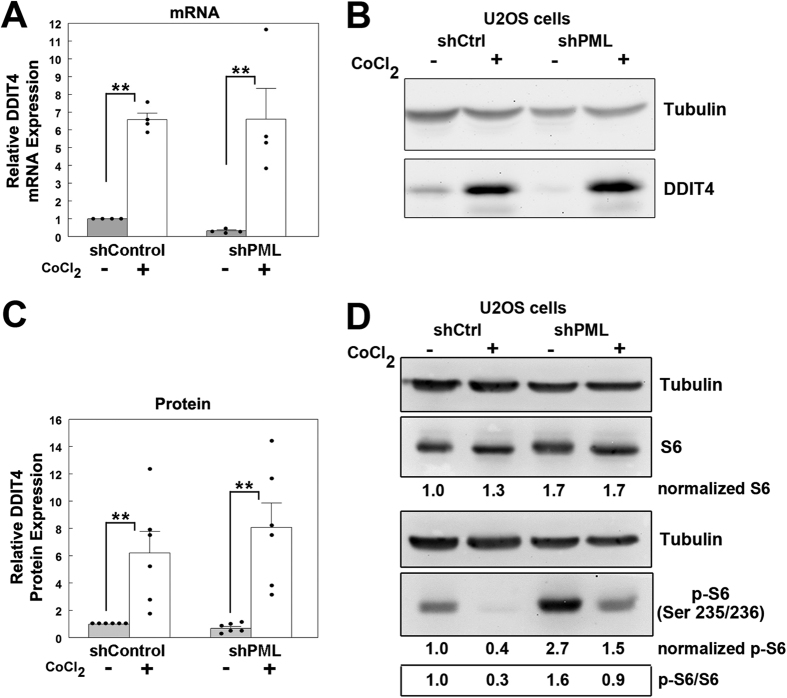
CoCl2-induced hypoxia-like stress upregulates DDIT4 expression independent of PML. (**A**) Quantitative PCR analysis of DDIT4 mRNA expression in shControl and shPML cells treated with 500 μm CoCl_2_ for 24 h (n = 4). (**B**) Western blot analysis of DDIT4 expression in shControl and shPML U2OS cells treated with 500 μm CoCl_2_ for 24 h. (**C**) Relative DDIT4 expression in CoCl_2_-treated U2OS from cells treated as in A (n = 6). (**D**) Western blot analysis and densitometry of S6 and phospho(Ser235/236) S6 levels in shControl and shPML U2OS cells treated with 500 μm CoCl_2_ for 24 h. Values in A and C represent the mean+/−SEM. *p-*values < 0.01 (**) or <0.05 (*) and n.s. = not significant. Full-length blots for this figure are presented in Supplementary Figure 5.

Having established that PML loss reduces basal DDIT gene expression, we next explored whether PML silencing affects the ability of cells to regulate DDIT4 protein expression in response to hypoxia-like, metabolic, and genotoxic stress. DDIT4 is highly upregulated in response to hypoxic stress, and PML can inhibit mTOR activation under hypoxic conditions^20^. Therefore, we also analyzed DDIT4 response in shPML cells to hypoxia-like conditions induced by cobalt chloride (CoCl_2_) treatment, which simulates hypoxic signaling through the stabilization of HIF1α and has been used to induce DDIT4 expression^24^. CoCl_2_ treatment significantly increased DDIT4 mRNA levels in both the shControl and shPML U2OS cells (*p* < 0.01; Fig. 5A). This increase was correlated with an increase in DDIT4 protein expression (Fig. 5B). When these protein changes were quantified, we identified a ~6-fold increase in CoCl_2_-treated shControl cells (Fig. 5C). CoCl_2_-treated shPML U2OS cells had a similarly robust upregulation of DDIT4, reaching levels similar to CoCl_2_-treated shControl cells (Fig. 5C), suggesting that PML loss does not substantially affect DDIT4 induction in response to hypoxia-like stress. To assess the functional consequence of DDIT4 upregulation, we again analyzed S6 phosphorylation as an indicator of mTOR activity (Fig. 5D). In both shControl and shPML cells, CoCl_2_ treatment resulted in decreased phosphorylation of S6, consistent with the observed increases in DDIT4 and its inhibitory activity on mTOR. However, although DDIT4 was robustly activated in both cell lines with CoCl_2_ (Fig. 2B), we did observe that the shPML cells retained some S6 phosphorylation in the presence of CoCl_2_ whereas phosphorylation of S6 was undetectable in the shControl cells. These results are consistent with a role of PML in directly inhibiting mTOR under hypoxic conditions^20^ rather than through regulation of DDIT4.

### Reduced basal DDIT4 expression with PML silencing indirectly limits DDIT4 upregulation following metformin and ionizing radiation treatment

**Figure 6:**
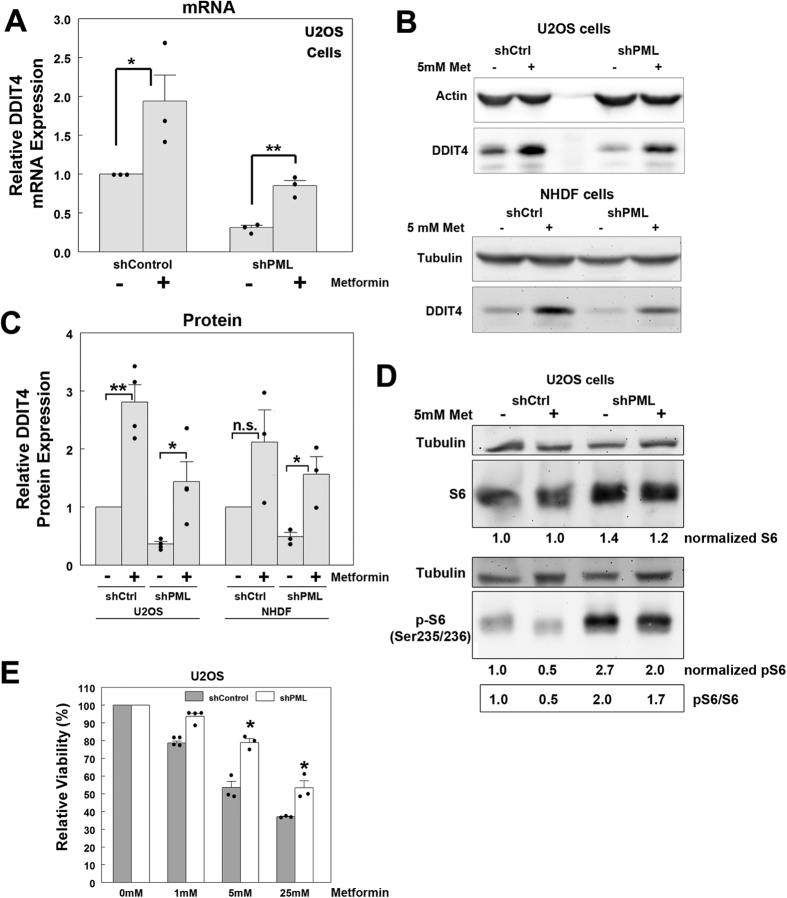
Metformin upregulates DDIT4 expression independent of PML. (**A**) Quantitative PCR analysis of DDIT4 mRNA expression in shControl and shPML cells treated with 5 mM metformin for 24 h (n = 3). (**B**) Western blot analysis of DDIT4 expression in shControl and shPML U2OS and NHDF cells treated with 5 mM metformin for 24 h. (**C**) Relative DDIT4 protein expression in metformin-treated U2OS (n = 4) and NHDF (n = 3) cells treated as in A. (**D**) Western blot analysis and densitometry of S6 and phospho(Ser235/236) S6 levels in shControl and shPML U2OS cells treated with 5 mM metformin for 24 h. (**E**) Relative cell viability for shControl and shPML cells treated with the indicated doses of metformin for 72 h (n = 3). For (**A**,**C** and **E**), values represent the mean+/−SEM. *p-*values < 0.01 (**) or <0.05 (*) and n.s. = not significant. Full-length blots for this figure are presented in Supplementary Figure 6.

Metformin is an inhibitor of oxidative phosphorylation and produces metabolic and oxidative and energy stress in treated cells^38^ which increases *DDIT4* expression in an AMPK-independent and p53-dependent manner, resulting in mTOR and growth inhibition^26^. Since PML is also implicated in p53-mediated transcriptional control^16^,^17^,^39^, we wanted to assess if PML loss could affect DDIT4 expression in response to metformin. Using quantitative real time PCR, we found that metformin treatment (5 mM) increased DDIT4 mRNA expression in both the shControl and shPML U2OS cells (Fig. 6A). This mRNA upregulation correlated with an increase in DDIT4 protein expression in U2OS cells as assessed by Western blotting (Fig. 6B). Similarly, metformin treatment also increased DDIT4 expression in the shControl and shPML NHDF cells (Fig. 6B). Quantification of the relative DDIT4 protein levels indicated significant (*p* < 0.05) DDIT4 upregulation with metformin-treatment in both the shControl and shPML U2OS cells (Fig. 6C). A similar trend was also observed in the NHDF cells (Fig. 6C). Thus, these data indicate that metformin-mediated increases in DDIT4 expression are not dependent on the presence of PML. However, the reduced basal levels of DDIT4 in untreated shPML cells results in reduced overall levels of DDIT4 following metformin treatment in shPML U2OS cells as compared to metformin-treated shControl cells (Fig. 6A and C). Finally, to assess the response of DDIT4 to genotoxic damage we used ionizing radiation (IR), which can induce DDIT4 in a p53-dependent manner^23^,^40^. Similar to metformin treatment, 8 Gy of IR induced DDIT4 expression in both shControl and shPML U2OS cells but the final level of DDIT4 protein was reduced in PML-depleted versus control cells (Fig. S2).

The differences in total DDIT4 after metformin treatment could result in less efficient inhibition of mTOR by DDIT4 in the shPML cells. We therefore also assessed the impact of metformin treatment on the activation of mTOR by monitoring phospho-S6 in shControl or shPML U2OS cells (Fig. 6D). In shControl cells, metformin caused a 50% reduction in phospho-S6, consistent with an increase in mTOR inhibition via increased DDIT4. In contrast, the untreated shPML cells showed increased phospho-S6 relative to untreated shControl cells, consistent with decrease DDIT4 levels in the shPML cells, and metformin treatment only reduced phospho-S6 by 15%. Although metformin caused a dose-dependent decrease in viability in both the U2OS shControl and shPML cells, the shPML cells were more resistant overall (Fig. 6E). Thus, loss of PML reduces the sensitivity of U2OS cells to metformin, and our data implicates dysregulation of DDIT4 as a possible mechanism promoting cell growth in metformin-treated cells that correlates with increased mTOR activity.

Together, our data support a model in which PML loss is associated with decreased basal DDIT4 protein expression but not the direct induction of DDIT4 in response to metabolic, hypoxic and genotoxic stress. However, PML loss and the associated reduction in basal DDIT4 expression might indirectly influence the overall response to metabolic (e.g. metformin) stress.

### PML and DDIT4 expression are positively correlated in several cancer cell lines and cancer types

Our data indicates that, under normal conditions, PML NBs play an important role in maintaining basal *DDIT4* gene expression and that PML loss can lead to dysregulation of DDIT4 expression and therefore inappropriate mTOR activity that could drive cancer growth and progression. Therefore, we predict that PML and DDIT4 expression should be positively correlated, where cancer cells with low *PML* have low *DDIT4* gene expression and vice versa. To test this hypothesis, we analyzed cancer genomics data available through cBioPortal (http://www.cbioportal.org) for mRNA expression correlations between *PML* and *DDIT4, BCL2* or *TAP1*. Our initial analysis of 967 cancer cell lines from the Broad Institute’s Cancer Cell Line Encyclopedia (http://www.broadinstitute.org/ccle)^41^ showed weak (Spearman’s *r* = 0.199) but significant (*p* < 0.01) positive correlation between *PML* and *DDIT4* gene expression (Table 1). In comparison, *PML* and *TAP1* showed a stronger correlation (Spearman’s *r* = 0.489, *p* < 0.01); and as predicted from cell lines studies, *PML* and *BCL2* gene expression were not significantly correlated (Spearman’s = 0.006, *p* = 0.854). These data are consistent with previous studies showing a positive correlation between PML and TAP1 and no correlation between BCL2 and PML expression^11^,^12^, and our immuno-RNA FISH data that indicated that PML NBs are more closely associated with actively transcribing *DDIT4* loci but not *BCL2* loci (Fig. 6).

**Figure 7:**
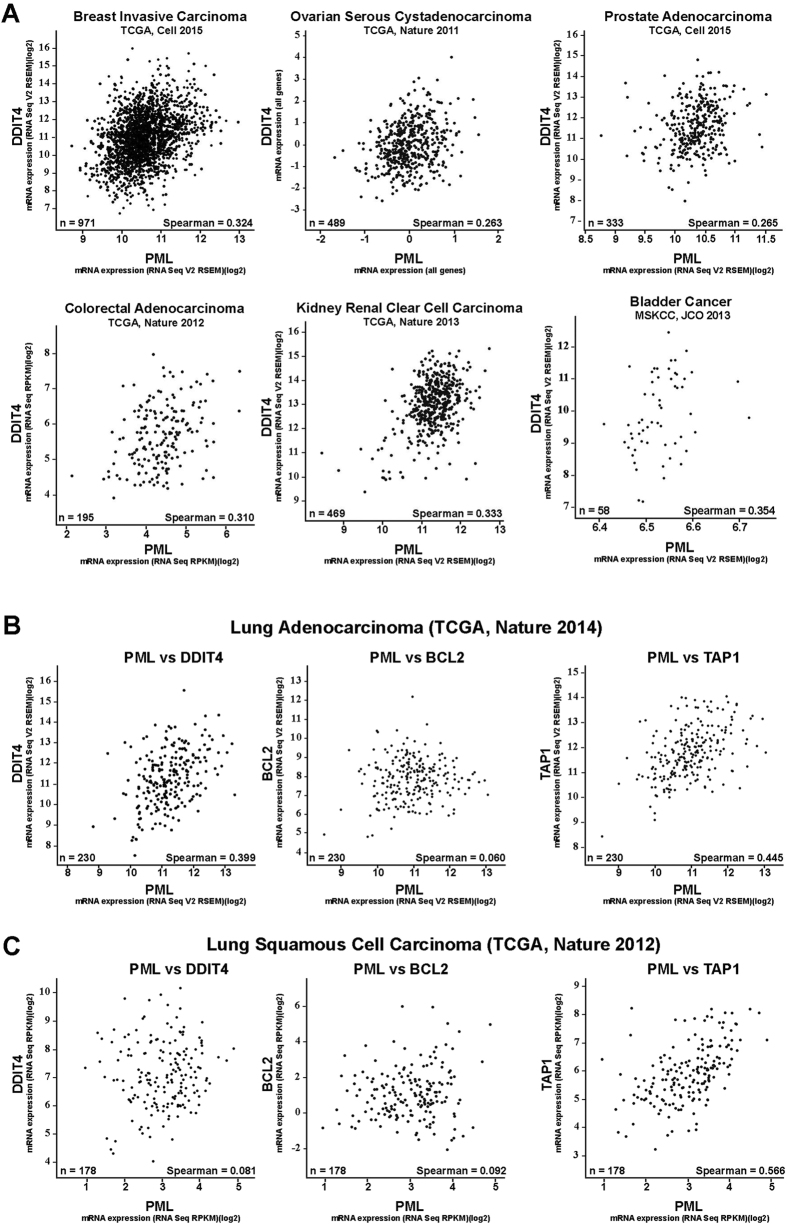
PML and DDIT4 expression is positively correlated in human cancers. Gene expression data from human cancers was analyzed using the cBioportal web interface and correlation plots with Spearman’s correlations are presented. (**A**) DDIT4 and PML mRNA expression correlation plots for human breast, ovarian, prostate, colorectal, kidney and bladder cancers. (**B**) PML mRNA expression data for 230 lung adenocarcinoma samples were correlated with DDIT4, BCL2 and TAP1 mRNA expression. (**C**) PML mRNA expression data for 178 lung squamous cell carcinoma samples were correlated with DDIT4, BCL2 and TAP1 mRNA expression.

We also assessed *PML* and *DDIT4* mRNA expression correlations by cancer cell tissue type and found significant (*p* < 0.05) and strong correlations (Spearman’s *r* = 0.275–0.345) in breast, ovarian and lung cancer cells (Table 1). Expression data for U2OS cells were also included in the CCLE osteosarcoma cell line data and, although the data was not significant (*p* = 0.130), *DDIT4* showed a positive correlation with *PML* (Spearman’s *r* = 0.312), consistent with our observations in this study. Analysis of other available TCGA gene expression data sets for human cancers accessible via the cBioPortal also identified cancers where *DDIT4* and *PML* expression were significantly (*p* < 0.05) and positively (Spearman’s *r* = 0.263–0.354) correlated, including breast^42^, ovarian^43^, prostate^44^, colorectal^45^, kidney^46^ bladder^47^ and lung^48^ cancers (Fig. 7A and B).

Since the strongest correlation between *DDIT4* and *PML* expression occurred in lung cancer derived cell lines (Table 1), and specifically lung adenocarcinoma (Spearman’s 0.399, *p* < 0.01, Fig. 7B), we extended our analysis to lung cancer subtypes. In addition to a positive correlation between *DDIT4* and *PML* mRNA expression in lung adenocarcinomas (Fig. 7B), *PML* and *TAP1* showed a similar positive correlation (Spearman’s *r* = 0.445, *p* < 0.01, Fig. 7B) and no correlation between *PML* and *BCL2* (Spearman’s *r* = 0.060, *p* = *0.365,* Fig. 7B). In contrast, *PML* and *DDIT4* showed no correlation (Spearman’s *r* = 0.081, *p* = 0.281) in lung squamous cell carcinomas (Fig. 7C)^49^; whereas, *PML* and *TAP1* were positively correlated (Spearman’s *r* = 0.566, *p* < 0.01). As expected, *PML* and *BCL2* were not correlated (Spearman’s *r* = 0.092, *p* = 0.219) in the lung squamous cell carcinoma samples. Thus, our analysis of TCGA and Broad cancer gene expression data sets revealed that *PML* and *DDIT4* are positively correlated in several human cancer types, particularly those that are known to have lower *PML* (i.e. lung, breast, prostate)^1^. Thus, these analyses suggest that PML likely plays a role in regulating *DDIT4* gene expression in multiple cell types and cancers of diverse tissue origin.

## Discussion

PML NBs are associated with transcriptionally active gene loci^12^; however, few genes are known that are specifically associated with and regulated by PML NBs. PML NBs are also involved in regulating cell fate decisions (e.g. apoptosis, senescence) following cellular stress (e.g. hypoxia, DNA damage), and loss of PML can promote cancer cell growth in part by activating mTOR^4^,^5^. In this study, we uncovered a novel mechanism by which PML loss can contribute to mTOR activation via the dysregulation of DDIT4, an inhibitor of mTOR. Silencing PML expression in both U2OS and NHDF cells resulted in decreased DDIT4 protein expression that correlated with increased mTOR activity (Fig. 1). The dysregulation of DDIT4 under basal conditions is, at least in part, due to altered *DDIT4* transcriptional regulation as indicated by reduced mRNA levels in PML-depleted cells (Fig. 2), and by the close association of actively transcribing *DDIT4* gene loci with PML NBs (i.e. within 1.1 μm, *p-*value < 0.0001; Fig. 4). PML loss did not appear to affect the upregulation of DDIT4 in response to hypoxia-like (CoCl_2_, Fig. 5), metabolic (metformin, Fig. 6), or genotoxic stress (IR, Fig. S2). However, the reduced levels of basal DDIT4 in the shPML cells were correlated with reduced overall levels of DDIT4 compared to shControl cells following metformin treatment. Thus, our study has identified DDIT4 as a novel PML NB-associated gene, which like *TAP1*^10^,^50^, may be regulated through its association with PML NBs.

How PML and PML NBs might regulate *DDIT4* gene expression is unclear but may involve coordination of active chromosomal domains at PML NBs and/or the post-translational modification (or reversible sequestration) of TFs and chromatin remodeling enzymes at PML NBs^14^. This is the most likely scenario given that PML is not known to act as a transcription factor or repressor directly by binding DNA. In addition, even though PML NBs make extensive contacts with chromatin at their periphery, the bodies themselves are devoid of nucleic acid^51^ and thus genes regulated by PML NB-association would never appear to colocalize but rather are always juxtaposed to the body^10^,^11^,^52^. Although beyond the scope of this work, identification of these TFs and chromatin enzymes is rather daunting given that the ENCODE ChIP-Seq data reveals over 60 transcriptional regulators that can associate with the DDIT4 promoter. However, we can speculate about likely candidates, of which there are several based on known association with PML and PML NBs in the literature. These include SP1, EP300, JUN/FOS, HIF1α and p53, to name just a few that might regulate DDIT4 basal and inducible-expression following cellular stress in a PML- or PML NB-dependent manner. For example, p53 and SP1 can associate with PML NBs and are known to regulate DDIT4 expression^15^,^23^,^27^,^53^ and could therefore contribute to the basal regulation of DDIT4.

In our studies analyzing *DDIT4* expression in response to metformin (Fig. 6) and IR (Fig. S2), the induction of *DDIT4* did not appear to be PML-dependent. However, the maximal level of DDIT4 protein achieved in PML-depleted U2OS cells following these stresses was reduced relative to treated control cells. PML NBs play a role in p53 activation, and *DDIT4* induction in response to metformin^26^ and IR is regulated by p53^40^. Since p53 silencing reduces basal DDIT4 expression^26^, we speculate that the basal regulation of *DDIT4* expression by p53 may be impaired in PML-depleted cells, and that this then leads to an attenuated *DDIT4* response to metformin- and IR-induced stress.

Finally, our study provides a new context in which to view changes in PML expression during cancer development and progression. PML is an important tumor suppressor whose expression is frequently reduced or lost in several cancers from diverse tissue types such as lung, prostate, colon and breast^1^. One previously uncharacterized consequence of the loss of PML, uncovered in this study, is a reduction of DDIT4 expression (Fig. 1). Our analysis of the TCGA cancer transcriptome datasets available through the cBio Portal (http://www.cbioportal.org/), revealed a positive correlation between PML and DDIT4 expression in several cancers, including breast, prostate, ovarian and lung cancers (Fig. 7). Based on our results in U2OS cells and our analysis of cancer transcriptomes, it is likely that reduced PML expression observed in these cancers is correlated with reduced DDIT4 expression, which in turn could promote mTOR activation and promote cancer cell growth. Thus, in addition to PML NB-mediated nuclear retention of mTOR and inactivation of AKT, we have uncovered a novel mechanism by which PML NB loss contributes to mTOR activation in cancer via dysregulation of DDIT4 gene expression.

## Materials and Methods

### Reagents and drug/chemical treatments

All chemicals were purchased from Sigma Aldrich unless otherwise indicated. Metformin and CoCl_2_ were dissolved in growth media and added to cells for 24 h unless otherwise indicated in the figure legends. For Western Blotting, CoCl_2_-treated cells were washed with PBS containing 500 μM CoCl_2_ to prevent reversal of drug response prior to cell lysis. Cycloheximide was added to growth media at 50 μg/ml.

### Cell lines

Low passage U2OS osteosarcoma cells (source: ATCC, HTB-96) were maintained in DMEM supplemented with 10% fetal bovine serum (FBS). TERT-immortalized normal human diploid fibroblasts (NHDF) were generated from GM05757 cells^54^, and maintained in α-MEM supplemented with 15% FBS. U2OS and NHDF cells were used to generate cell lines expressing lentivirus-delivered shRNAs targeting luciferase (shCtrl-Luc, GTGCGTTGCTAGTACCAAC) or exon 4 of PML (shPML-A, AGATGCAGCTGTATCCAAG) which is common to all major PML isoforms^28^. Similarly, U2OS cells were used to generate cell lines expressing lentivirus-delivered control non-targeting shRNA (shCtrl-NT, Dharmacon, #RHS 4346, sequence: ATCTCGCTTGGGCGAGAGTAAG) or shRNA targeting PML exon 6 which is common to all nuclear PML isoforms (shPML-B, Dharmacon, Clone ID: V3LHS_638440, sequence: CCATCTTGATGACCTTCCT). All cells were maintained at 37 °C and 5% CO_2._ Media for shControl and shPML cells was supplemented with 1 μg/ml of puromycin.

### Western Blotting

Antibodies used for Western blotting analysis were: rabbit anti-PML (Bethyl Laboratories, A301–167A, 1:2000), mouse anti-PML (E-11, Santa Cruz Biotechnology, sc377390, 1:2000), mouse anti-Actin (Sigma, A 5316, 1:5000), rabbit anti-Tubulin (Santa Cruz Biotechnology, sc-9104, 1:5000), rabbit anti-DDIT4 (Proteintech, 10638–1-AP, 1:500), rabbit anti-ribosomal subunit S6 (Cell Signalling Technology, cat# 2217, 1:1000), and rabbit anti-phospho S235/236 ribosomal subunit S6 (Cell Signalling Technology, cat# 4858, 1:1000).

Cells were lysed with RIPA buffer (Sigma, cat#R0278) containing protease inhibitors (Sigma, cat#P8340) and subjected to SDS-PAGE previous described^34^. Lysates prepared for analysis of phospho-S6 contained 50 mM NaF and 100 μM NaOV_3_. Indicated primary antibodies were incubated overnight at 4 °C and peroxidase conjugated secondary antibodies were incubated at RT for 45 min. Immunoblotted membranes were developed with Bio-Rad Clarity Western substrate kit (Bio-Rad, cat#170–5061) and imaged on a VersaDoc MP 4000 (Bio-Rad). Images were processed in QuantityOne (Bio-Rad) and Adobe Photoshop CS5 (Adobe) using only linear adjustments (e.g. brightness and contrast) and noise filtering (e.g. despeckle) to reduce camera noise.

### AlamarBlue Viability Assay

Cell viability and metformin sensitivity of shControl and shPML U2OS cells was measured using the AlamarBlue assay (Life Technologies Ltd., cat#DAL1100) following the manufactures instructions. Briefly, 1500 cells were seeded in 96-well plates and grown for 24, 48, and 72 h. AlamarBlue reagent was added to 4 wells per condition at each time point, incubated for 2–4 h at 37 °C and the amount of dye reduction was quantified by measuring the absorbance at 590 nm using an Infinite 200 Pro microplate reader (Tecan US Inc., Morrisville, NC). Metformin (1 mM, 5 mM, 25 mM) was added to cells 24 h after plating and AlamarBlue was added for 4 h at 48 h post-metformin treatment.

### Translation State Array Analysis

Polysome profiling was performed essentially as previously described^36^. Cytoplasmic extracts containing polysomes from shControl and shPML U2OS cells were loaded onto a 10–50% sucrose gradient and polysomes were separated by ultracentrifugation in a Beckman SW41 rotor at 36 000 rpm for 1 h 50 min, 4 °C. The sucrose gradients were fractionated and absorbance at 254 nm was determined. RNA was harvested from fractions containing the high molecular weight polysomes and the isolated RNA was pooled and concentrated by ethanol precipitation for microarray analysis. Total cellular RNA was also harvested in parallel for microarray analysis.

The quality of RNA samples was assessed using the Experion Bioanalyzer system (Bio-Rad) and had RNA Quality Index values greater than 9. Each RNA sample (1 μg) was amplified using the Amino Allyl MessageAmp II aRNA amplification kit and labeled with AlexaFluor 555 or 647 (Life Technologies). Quantity and quality of amplified aRNA was assessed using a Nanodrop spectrophotometer and the Experion Bioanalyzer. Samples were compared in a dye swap experiment, with 3 μg of each labeled, fragmented aRNA (6 μg total per slide) hybridized to proprietary human cDNA microarray slides. These arrays consist of roughly 35 000 spots, representing roughly 17 000 different 50-mer oligonucleotides spotted in duplicate on Nexterion-E epoxy microarray slides (Schott).

Hybridizations were performed in Ambion SlideHyb #2 buffer (Life Technologies) at 42 °C for 16 h using the automated TECAN-4800 Hybridization station (TECAN). Hybridized slides were scanned at 10 μm resolution using an Axon GenePix 4200AL scanner (Molecular Devices, Sunnyvale, CA) and gridded using SpotReader (Niles Scientific). Calibration of spot rejection was done by visual inspection of gridded images and scatter plots of M = log(532/635) versus A = log(532) + log(635), with special attention being paid to outliers. A GPR file was generated and processed to flag spots with a signal to noise ratio <5.

Analysis was performed with Acuity 4.0 (Axon Instruments). The data were normalized using Lowess and the relative mRNA abundances for DDIT4 were determined for 3 independent experiments. Student’s t-tests were used to calculate *p*-values.

### QRT-PCR

U2OS shControl and shPML cells at 70% confluence in 10 cm dishes were directly solubilized in TRIzol reagent (Life Technologies, cat#15596–026), followed by total RNA extraction and purification. cDNA was generated from 1 μg RNA using the iScript Reverse Transcription Supermix protocol (Bio-Rad, cat#170–8841). QRT-PCR for PML, DDIT4, BCL2, TAP1, and reference genes HMBS and RAC1 were conducted using SsoAdvanced Universal SYBR Green Supermix (Bio-Rad, cat#172–5271) on a CFX96 Touch Real-Time PCR Detection System (Bio-Rad). Reference gene selection (HMBS and RAC1, Mean CV = 0.0986, Mean M-value = 0.2845), primer optimization, and experimental data collection were conducted in 4 reactions for 3 biological replicates and complied with MIQE guidelines^55^. Quantitative data analysis was performed on CFX Manager Software (Bio-Rad, v3.1). Primers were designed using Primer 3-Blast (Primer-Blast, NCBI, Bethesda, MD) and are listed 5′-3′: PML Forward (ATGGCTTCGACGAGTTCAAGG), PML Reverse (TACAGCTGCATCTTTCCCCTG), DDIT4 Forward (GGTTTGACCGCTCCACGAG), DDIT4 Reverse (ATCCAGGTAAGCCGTGTCTTC), BCL2 Forward (CAGGATAACGGAGGCTGGGATG), BCL2 Reverse (GGGCCAAACTGAGCAGAGTC), TAP1 Forward (TTACACTTGGAGGGCCTTGTC), TAP1 Reverse (GGTGAATGTCAGCCCCTGTAG), HMBS Forward (GTAACGGCAATGCGGCTG), HMBS Reverse (ACACTGTCCGTCTGTATGCG), RAC1 Forward (CACTGTCCCAACACTCCCAT), RAC1 Reverse (GACCCTGCGGATAGGTGATG).

### Luciferase Assay

A 1683 bp fragment of the DDIT4 promoter region was amplified from U2OS genomic DNA (forward 5′-CTCCAACAAGCGACAGAGCTTG-3′; reverse 5′-GAGAACTGCTAAGACAAGTGCGTC-3′) and cloned into the promoterless vector PGL4.14 encoding Firefly luciferase (Promega). For promoter assays, empty PGL4.14 vector (pLuc) or that containing the DDIT4 promoter (pLuc-DDIT4) were transfected into shPML and shControl U2OS cells using Lipofectamine 2000 (Life Technologies) using Renilla luciferase (HSV-TK-Renilla) as a normalization control for transfection. After 24 h cells were lysed in 1X passive lysis buffer (Promega) and Firefly and Renilla expression were determined using the Dual-Reporter Luciferase Reporter System (Promega) and a Promega GloMax lumminometer.

### 3D-ImmunoFISH

For 3D-ImmunoFISH, the following Spectrum Orange-labelled BAC probes were obtained from The Centre for Applied Genomics (TCAG, The Hospital for Sick Children, Toronto): DDIT4, RP11–692J22; TAP1, RP11–10A19; PML, RP11–185E17; BCL2, RP11–160M23. Our 3D-immunoFISH protocol was adapted from Cremer *et al*.^56^ and Ching *et al*.^11^. Briefly, DNA hybridization was conducted on formaldehyde fixed and permeabilized U2OS cells grown on glass slides using the labelled BAC probes. Cells were then immunostained with rabbit anti-PML antibody (Bethyl, 1:1000) and labelled with goat anti-rabbit alexaFluor 488-conjugated secondary antibody (1:800). Cells were then incubated with DAPI to visualize DNA and mounted with coverslips using fluorescent mounting medium (Dako).

Fluorescent images were acquired on a customized Zeiss Cell Observer inverted microscope (Intelligent Imaging Innovations) equipped with a CoolSNAP HQ2 CCD camera (Photometrics) using a 1.4 NA 63 × oil-immersion lens and captured using Slidebook 5.1 software (Intelligent Imaging Innovations). Image stacks were exported to Imaris 7.2 software (Bitplane) for further analysis. Distances between PML NBs and the FISH probes were determined in 3D after minimal threshold adjustments to define the Spectrum Orange labelled probes and PML NB immuno-signal followed by semi-automated image processing, which included manual validation that detected centroid positions were accurately defined to PML NBs and most intense FISH probe signal. The Euclidian distance between the centroid of fluorescence of defined FISH probe signals and closest PML body were then calculated for each DAPI signal-defined nucleus and exported to Excel (Microsoft) for analysis. At least 50 nuclei were analyzed for each FISH probe to generate mean distances between a PML NBs and a given FISH signal for each FISH probe.

### RNA-immunoFISH

RNA FISH probe generation - PCR reactions to clone DNA fragments were generated using the following primers pairs: DDIT4 (forward 5′-CTCCGGCACTCTGAGTTCATC-3′ and reverse 5′-GACACCCACCCCTTCCTACTC-3′) and BCL2 (forward 5′-CTTCAACGACGGTTTATTTGCTGTC-3′ and reverse 5′-TATGCTGCAGATTGAAAAGTGCAAA-3′). Amplicons were gel purified and cloned using a pGEM T-easy cloning kit (Promega), sequenced and subsequently used for RNA probe generation. Briefly, 10 μg of linearized probe DNA was used for *in vitro* transcription with T7 RNA polymerase (New England Biolabs) following manufacturer’s instructions. To generate single stranded cDNA probes for each gene, 5 μg of purified RNAs were mixed with 1.5 μl of 5 μg/μl random hexamers. RNAs were denatured at 65 °C for 5 min and cooled immediately on ice to bind hexamers. After cooling, 4 μl of 5X first strand buffer, 2 μl 100 mM DDT and 7.5 μl of dNTP mix (0.7 mM biotin-16-dUTP, 1.3 mM dTTP, 2 mM dATP, 2 mM dCTP, 2 mM dGTP) were added. The reaction was heated to 42 °C for 2 min before the addition of 400 U of Superscript III (Invitrogen). The reaction mix was incubated at 42 °C for 90 min. RNA was removed from the reaction using 2 μl 4 M NaOH and incubation at 37 °C for 30 min. The reaction was neutralized using 5 μl 1 M Tris-HCl, pH 7.5 Probe was precipitated using ethanol sodium acetate precipitation.

The protocol used for FISH was modified from the Turbo-FISH protocol described by ref. 57. Precipitated probe was re-suspended in Denhart’s solution and 50 mM NaH2PO4/Na2HPO4. U2OS cells grown on coverslips were fixed in 4% formaldehyde/PBS (10 min, RT), followed by permeabilization in 0.5% triton-X/PBS (10 min, RT). Coverslips were incubated in pre-hybridization solution (2 X SSC, 10% formamide) for 30 min, followed by incubation with 15 μl of hybridization solution containing 300–500 ng of biotinylated probe. Slides were incubated for 1 h at 37 °C, then washed 3 × 5 min in PBS. Biotinylated probes were detected using a triple sandwich of streptavadin-Cy3 (1:200; Jackson Scientific, Cat #:#: 016-160-084), goat anti streptavidin-biotin, (1:200; Vector Laboratories, Cat #: Ba-0500) followed by incubation in streptavadin-Cy3 (1:200). PML protein was detected in combination with FISH using a rabbit anti-PML antibody (1:200, Cedarlane, Cat #: A301–167A) followed by incubation with donkey anti-rabbit A488 (1:200, Cedarlane, Cat #: 711-145-152). DNA was visualized with Hoechst 33342 dye (Invitrogen, Cat #: h3570). Images were acquired on a Nikon Eclipse Lv100 microscope equipped with a Nikon C2 CCD camera, using Nikon NIS Elements software. Images were processed using Adobe Photoshop CS 6.

### RNA-Sequencing

For RNA sequencing and differential expression analysis, shControl and shPML U2OS cells were grown to 80% confluence in 10 cm dishes and prepared for Illumina HiSeq sequencing as previously described^58^. Sequencing reads (GEO accession #GSE94126) were mapped using TopHat (version 2.0.0) to the known transcriptome (-G option; Gencode version 7 annotations) for the human reference genome (hg19) using default parameters^59^. Cufflinks (version 2.0.2) was used to quantify gene and isoform expression for known transcripts (-G option; Gencode version 7 annotations) using the default parameters^60^. Only uniquely aligned reads were used for expression quantification. Pairwise differential gene expression analysis was performed using Cuffdiff^61^ with the default parameters.

### Bioinformatics

Analysis of transcription factor ChIP-Seq data from ENCODE^62^ was conducted through the UCSC genome browser web interface (https://genome.ucsc.edu)^63^,^64^,^65^. Using the human reference genome (hg19)^66^, the ENCODE transcription factor ChIP-Seq peaks for PML^67^,^68^,^69^ that mapped to the promoter region of DDIT4 (RefSeq: NM_019058.2) were identified. The genomic positions, DNA sequences and ChIP-Seq signal scores were used for analysis and figure generation.

PML and DDIT4 gene expression data from multiple cancer datasets (e.g. TCGA https://tcga-data.nci.nih.gov/tcga/, Broad CCLE) available through cBioPortal (http://www.cbioportal.org/)^70^,^71^ was analyzed using custom scripts (https://github.com/dgaston/cbioportal-tools) to extract gene expression values, calculate Spearman’s correlation coefficients, and test for significance. Spearman’s correlation coefficient and *p*-values were calculated using the python scipy package (http://www.scipy.org/) with the scipy.sstats.pearmanr method and exported to Excel. DDIT4 and PML mRNA expression correlation plots for selected cancer data sets were generated in the cBioPortal web interface, exported to Adobe Photoshop, converted to greyscale and labelled for publication.

## Additional Information

**How to cite this article:** Salsman, J. *et al*. PML nuclear bodies contribute to the basal expression of the mTOR inhibitor DDIT4. *Sci. Rep.* **7**, 45038; doi: 10.1038/srep45038 (2017).

**Publisher's note:** Springer Nature remains neutral with regard to jurisdictional claims in published maps and institutional affiliations.

## Supplementary Material

Supplementary Information

## Figures and Tables

**Table 1:** DDIT4 and PML gene expression Spearman’s correlations from Cancer Cell Line Encyclopedia (Novartis/Broad, Nature 2012).

Cancer Type	II	Spearman’s	p-value
**All**	**967**	**0.199**	**0.000**
**Lung**	**174**	**0.345**	**0.000**
**Ovarian**	**50**	**0.305**	**0.031**
**Breast**	**58**	**0.275**	**0.036**
Biliary Tract	7	0.679	0.094
Soft Tissue	20	0.376	0.102
Upper Digestive Tract	30	0.297	0.111
***Bone***	***25***	***0.312***	***0.130***
Pleural	10	0.491	0.150
Urinary Tract	24	−0.276	0.192
Liver	27	0.232	0.244
Thyroid	12	0.364	0.245
Haematopoietic and Lyymphoid Tissue	178	0.081	0.285
Pancreatic	44	0.153	0.321
Nervous System	52	−0.128	0.364
Kidney	22	−0.191	0.393
Endometrial	25	−0.171	0.414
Skin	60	−0.098	0.457
Autonomic Ganglia	17	−0.169	0.516
Oesophageal	25	0.095	0.650
Prostate	7	−0.179	0.702
Large Intestine	57	−0.044	0.744
Stomach	38	0.038	0.822
Small Intestine	1	nan	nan
Salivary Gland	2	1.000	nan
