# Supplementary material for: PML nuclear bodies contribute to the basal expression of the mTOR inhibitor DDIT4
Source: Sci Rep. 2017 Mar 23;7:45038. doi: 10.1038/srep45038 (PMC5362932; doi:10.1038/srep45038)
Supplement: Supplementary Information [file srep45038-s1.pdf]

Supplemental Information

PML nuclear bodies contribute to the basal expression of the mTOR inhibitor DDIT4

Jayme Salsman[1], Alex Stathakis[1], Ellen Parker[1], Dudley Chung[1], Livia E. Anthes[1], Kara L. Koskowich[1], Sara Lahsaee[1], Daniel Gaston[1,2] Kimberly R. Kukurba[3], Kevin S. Smith[3], Ian C. Chute[4], Daniel Léger[4], Laura D. Frost[4], Stephen B. Montgomery[3], Stephen M. Lewis[4,5,6,7], Christopher Eskiw[8] & Graham Dellaire[1,9,10]

1. Department of Pathology, Dalhousie University, Halifax, Nova Scotia, B3H 4R2, Canada.
2. Department of Pathology and Laboratory Medicine, Nova Scotia Health Authority, Halifax, Nova Scotia, B3H 1V8, Canada.
3. Departments of Genetics and Pathology,Stanford University, Stanford, California, 94305, USA.
4. Atlantic Cancer Research Institute, Moncton, New Brunswick, E1C 8X3, Canada.
5. Department of Microbiology & Immunology, Dalhousie University, Halifax Nova Scotia, B3H 4R2 Canada.
6. Department of Chemistry & Biochemistry, Université de Moncton, Moncton, New Brunswick, E1A 3E9, Canada.
7. Department of Biology, University of New Brunswick, Saint John, New Brunswick, E2L 4L5, Canada.
8. Department of Department of Food and Bioproduct Sciences, University of Saskatchewan, Saskatoon, Saskatchewan, S7N5A8, Canada.
9. Department of Biochemistry & Molecular Biology, Dalhousie University, Halifax, Nova Scotia, B3H 4R2, Canada.
10. Beatrice Hunter Cancer Research Institute, Halifax, Nova Scotia, B3H 4R2, Canada

* To whom correspondence should be addressed. Tel: (902) 494-4730; Fax: (902) 494-2519; Email: dellaire@dal.ca

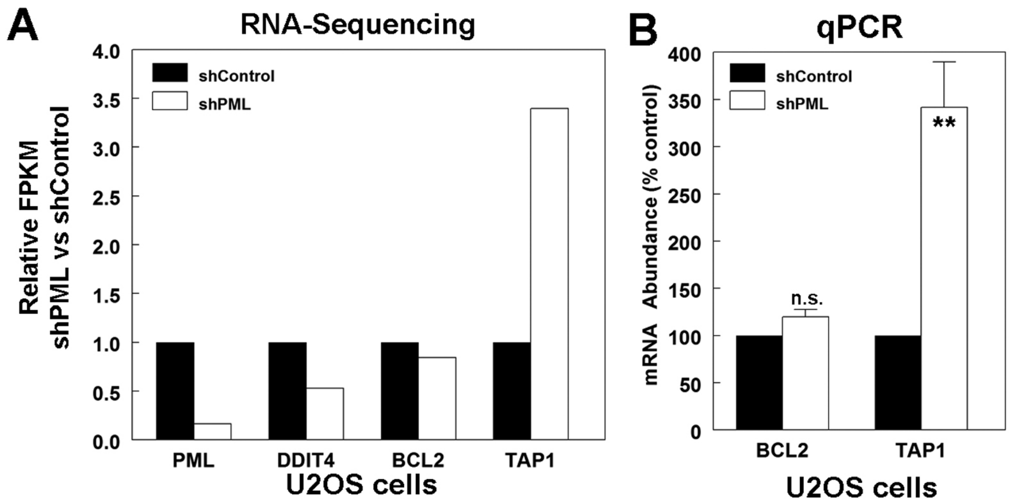

Supplemental Figure 1. PML, DDIT4, BCL2 and TAP1 gene expression in U2OS cells. (A) Expression values for DDIT4, PML, BCL2 and TAP1 genes in shPML U2OS relative to shControl cells as determined by RNA-sequencing. (B) Quantitative PCR was used to determine BCL2 and TAP1 mRNA abundance in U2OS shPML cells relative to shControl cells (n=3). Values in graphs represent the mean +/- SEM. Asterisks indicate *p*-value < 0.01 of student's t-tests. n.s. = not significant.

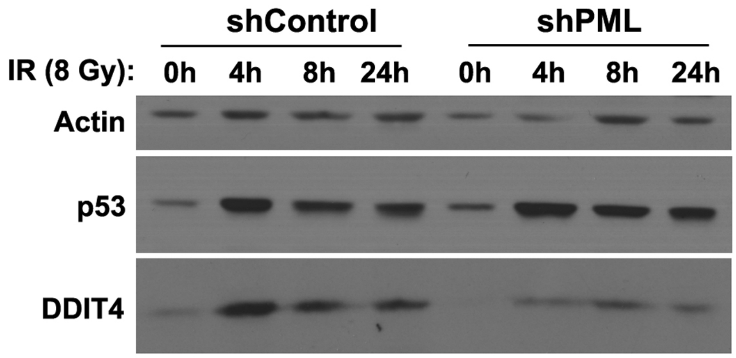

Supplemental Figure 2. PML loss limits DDIT4 response to ionizing radiation. Western blot analysis of DDIT4 and p53 expression in shControl and shPML U2OS cells treated with 8 Gy ionizing radiation (IR) and analyzed at the indicated times. Increased p53 with IR indicates activation of the DNA damage response. Total DDIT4 protein levels were lower in shPML cells following IR treatment at all time points.

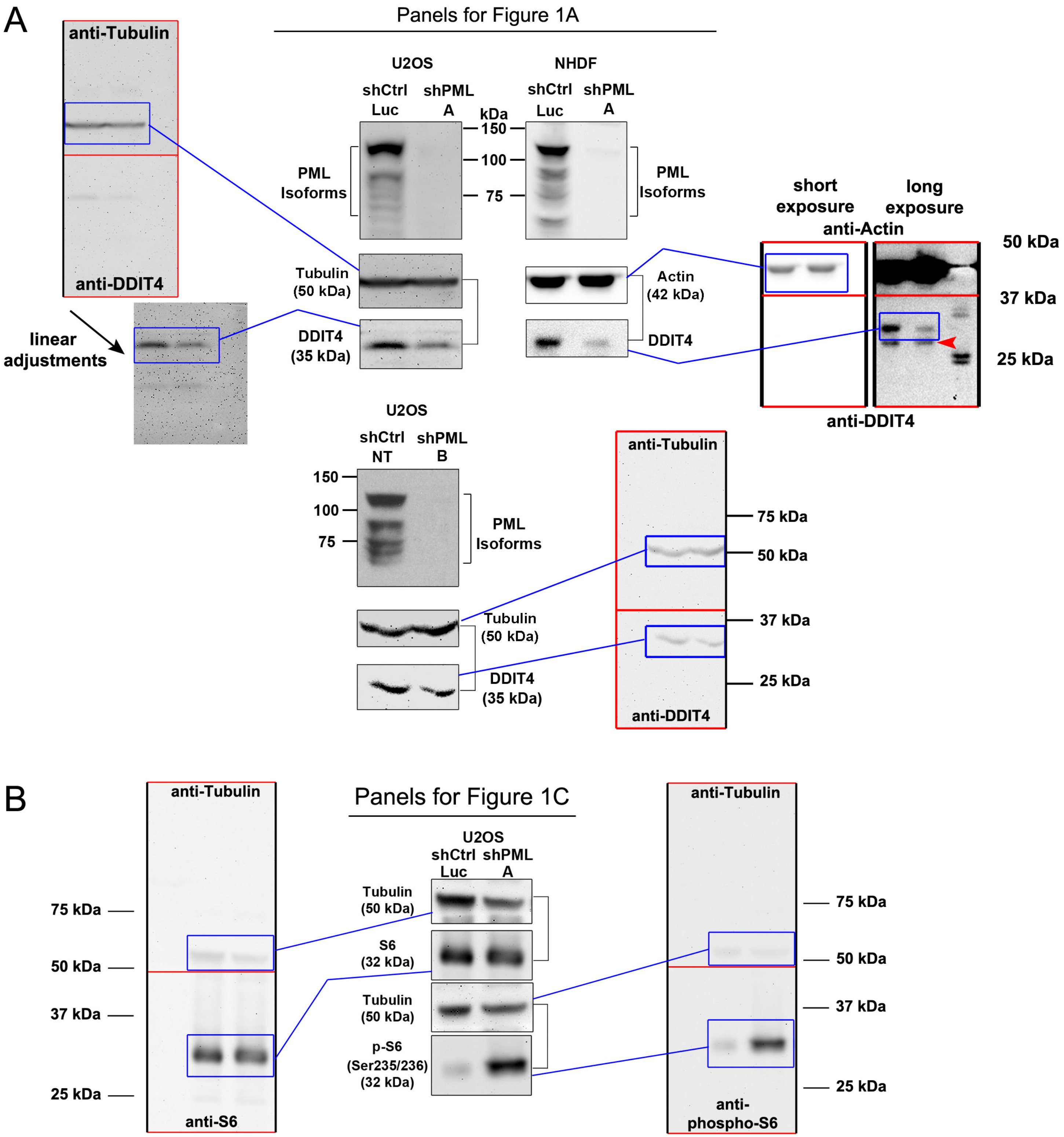

Supplemental Figure 3. Original Western blots for Figures 1A and 1C. (A) Full-length Western blots of DDIT4 expression in U2OS and NHDF cells with PML silencing presented in figure 1A. Membranes for PML blots were cut near the 50 kDa marker and are presented full-length in figure 1A. (B) Full length Western blots of S6 and phospho-S6 expression in U2OS shControl and shPML cells used to generate figure 1C. Membranes containing transferred protein were cut along the red lines and Western blotted for the indicated proteins. All gel images were acquired on a Versadoc gel imager. Empty lanes and irrelevant lanes from the original blots were cropped as indicated by black lines. Blue lines and boxes represent data used to generate figure panels as indicated. The red arrow indicates non-specific bands in the DDIT4 blot.

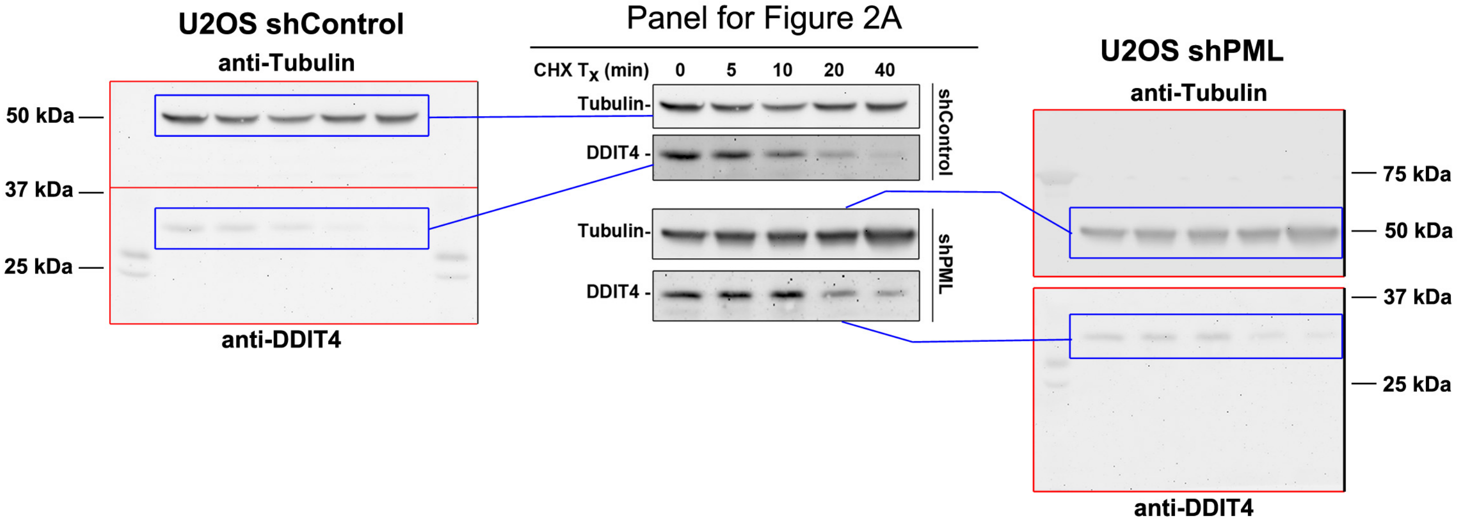

Supplemental Figure 4. Original Western blots for Figure 2A. Full-length Western blots of DDIT4 expression in cycloheximide-treated U2OS shControl and shPML cells presented in figure 2A. Membranes containing transferred protein were cut along the red lines and Western blotted for the indicated proteins. All gel images were acquired on a Versadoc gel imager. Empty lanes and irrelevant lanes from the original blots were cropped as indicated by black border lines. Blue lines and boxes represent data used to generate figure panels as indicated.

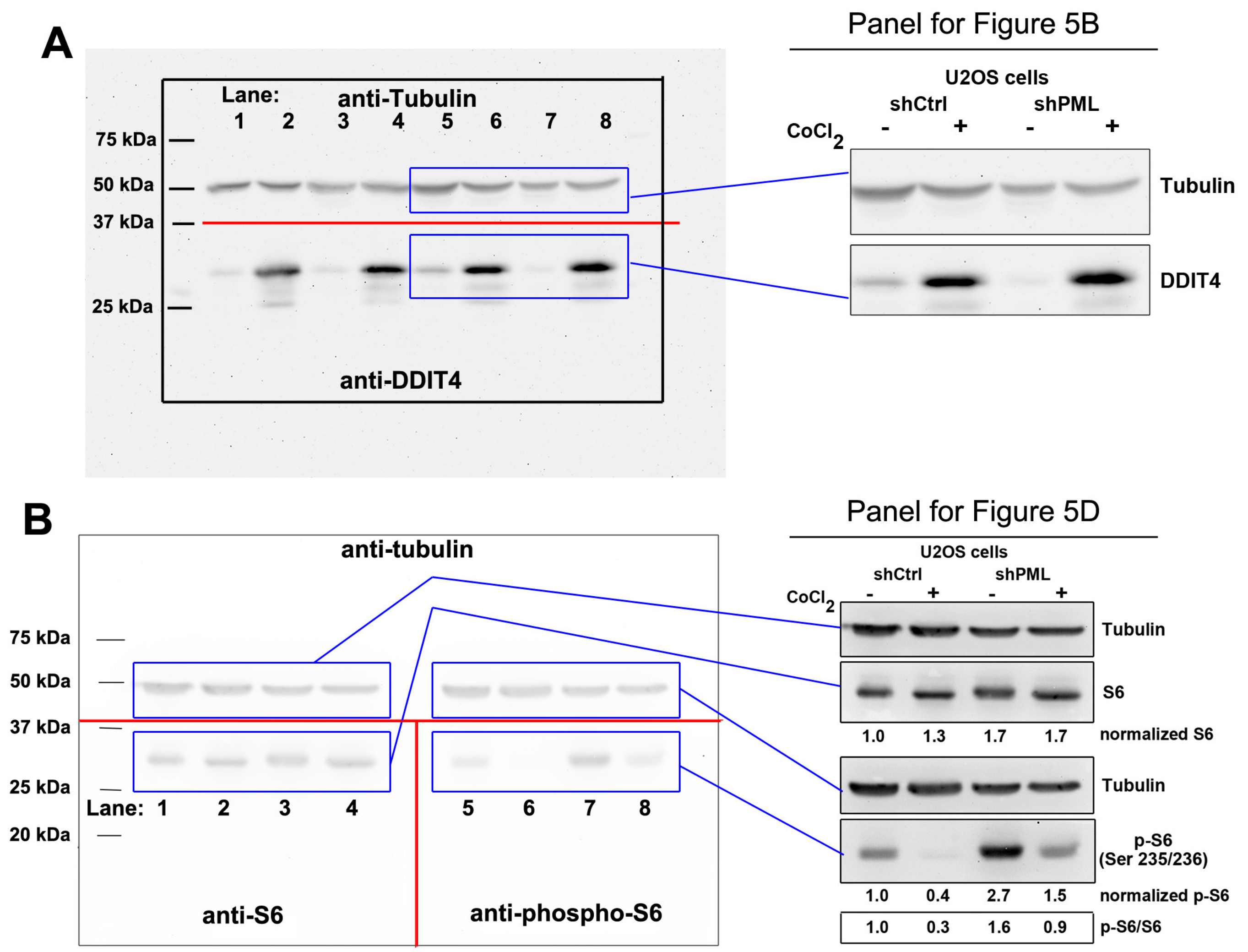

Supplemental Figure 5. Original Western blots used to generate figures 5B and 5D. (A) Full-length Western blot used to generate figure 5B. Lanes 1-4 and 5-8 are biological replicates run in parallel and the menbrane containing the transferred proteins was cut along the red lines, Western blotted for the indicated proteins and reassembled before imaging on a Versadoc gel imager. Lanes 5-8 were used to construct figure 5B as indicated with blue boxes and lines. (B) Full-length Western blot used to generate figure 5D. Lanes 1-4 and 5-8 represent identical samples that were run in duplicate on the same gel.The membrane containing the transferred proteins was cut along the red lines, Western blotted for the indicated proteins and reassembled before imaging on a Versadoc gel imager. Figure 5D was constructed as indicated with blue boxes and lines.

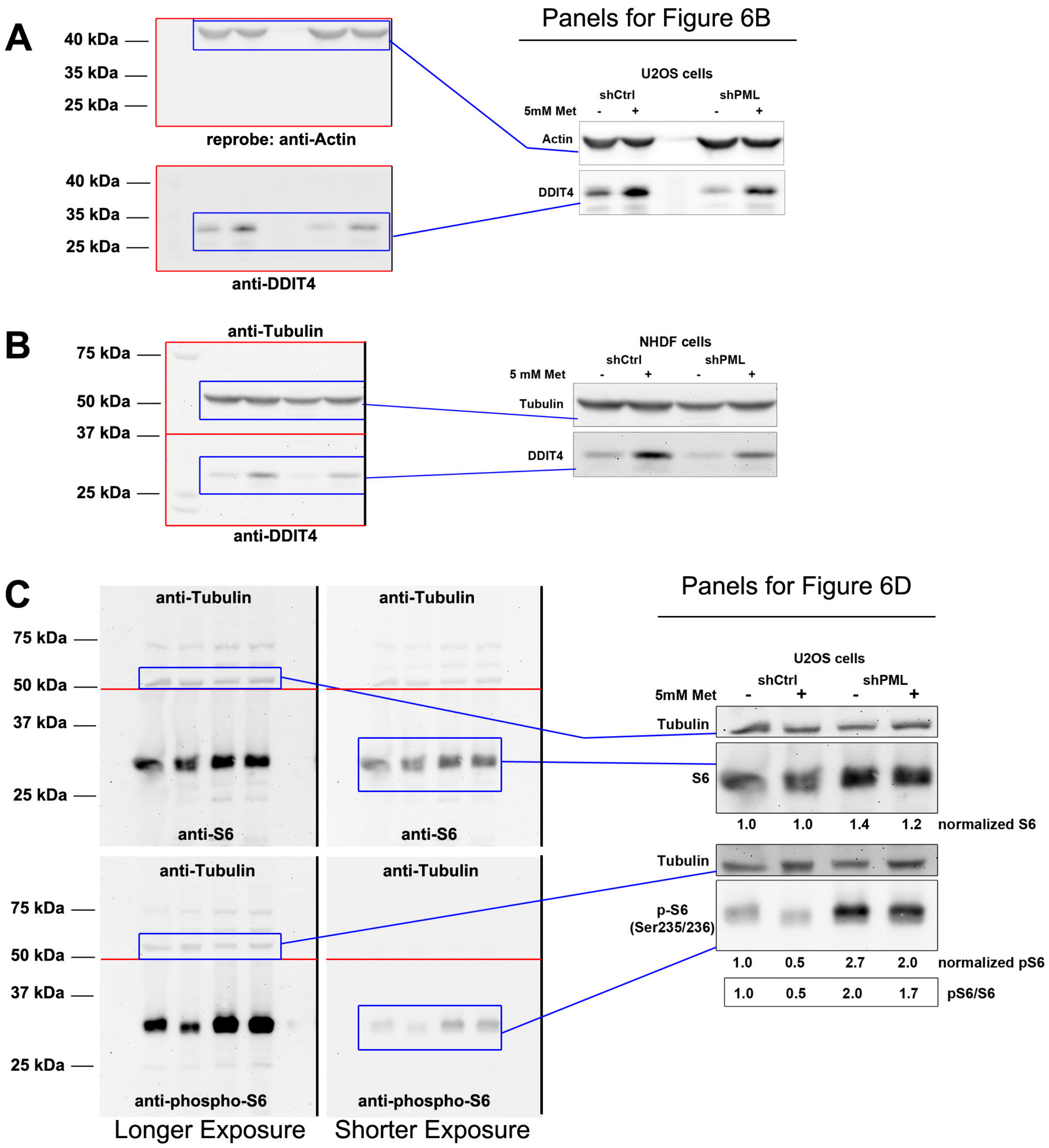

Supplemental Figure 6. Original Western blots for Figure 6B and D. Full-Length Western blots used to generate the U2OS (A) and NHDF (B) panels of figure 6B and Figure 6D (C). The membrane containing transferred protein was cut along the red lines and Western blotted for the indicated proteins. All gel images were acquired on a Versadoc gel imager. Empty and irrelevant lanes from the original blots were cropped as indicated by black border lines. Blue lines and boxes represent data used to generate figure panels as indicated.
